# Study on the High-Speed Penetration and Shear Failure Mechanism of Beach Sand

**DOI:** 10.3390/ma18091922

**Published:** 2025-04-24

**Authors:** Jianzhong Zhu, Jiangquan Li, Yun Kong, Mingming Dong, Yuqiong Li, Meng Zou

**Affiliations:** 1 Tianjin Key Laboratory for Advanced Mechatronic System Design and Intelligent Control, School of Mechanical Engineering, Tianjin University of Technology, Tianjin 300384, China; 2 National Demonstration Center for Experimental Mechanical and Electrical Engineering Education, Tianjin University of Technology, Tianjin 300384, China; 3 Key Laboratory for Bionics Engineering of Education Ministry, Jilin University, Changchun 130022, China; 4 School of Mechanical Engineering, Beijing Institute of Technology, Beijing 100081, China; 5 Key Laboratory for Mechanics in Fluid Solid Coupling Systems, Institute of Mechanics, Chinese Academy of Sciences, Beijing 100190, China; 6 State Key Laboratory of Nonlinear Mechanics, Institute of Mechanics, Chinese Academy of Sciences, Beijing 100190, China; 7 Guangdong Aerospace Research Academy, Nansha, Guangzhou 511458, China

**Keywords:** failure mechanism, high speed, beach sand

## Abstract

In this paper, the contact parameters of beach sand are calibrated based on the discrete element method and the optimal design method, and the obtained parameters by calibration are used as input for the angle of repose simulation. The relative error between angle of repose simulation results and experimental results is 3.27%. Based on the penetration and shear tests, simulation models were constructed to study the pressure shear failure mechanism of beach sand under high-speed conditions. The high-speed penetration simulation shows that with the increase of the penetration rate, the influence area of the sinkage plate gradually increases, and the stress of sand particles also increases. When the penetration rate increased from 0.5 m/s to 8 m/s, the pressure on the plate increased 12.7 times, indicating that the bearing capacity of the sand increased significantly with the increase of the penetration rate. The high-speed shear simulation shows that in the stable shear stage, the average shear torque initially increases slightly with the increase of speed, and then decreases significantly when the speed exceeds 4 m/s. This is because as the shear rate increases, the disturbance of the soil by the shear plate increases, the velocity of soil particles increases, resulting in a decrease in the number of soil particles in contact with the shear plate, thereby reducing the shear torque.

## 1. Introduction

Off-road vehicles are often used to complete a variety of tasks such as transporting equipment, and personnel, and need to maintain good maneuverability in a variety of complex terrain environments [1,2,3,4]. Since the coastal areas are mostly covered by soft sand, the mobility of vehicles on the beach sand has become a key factor restricting the vehicles from crossing the sea and land. How to effectively evaluate and improve the off-road maneuverability of vehicles has become an urgent problem that needs to be solved in the field of current off-road vehicles research [5,6,7].

The Discrete Element Method (DEM) is a numerical solution method based on the principle of discontinuity proposed by Cundall and Strack in 1971 [8]. The original intention of the method is to solve related problems of rock mechanics. In recent years, DEM has been widely used to analyze the interaction between vehicles and soil [9,10,11,12,13].

The modeling of interaction between different particles and between particles and boundaries should consider elasticity, viscosity, plasticity, friction, etc. The Hertz–Mindlin (no-slip) model is suitable for cases where there is no geometric viscosity between particles or the viscosity is negligible [14]. To consider the adhesive effect of contact regions, Johnson, Kendall, and Roberts proposed the theory of adhesive contact (JKR) [15]. The Hysteretic Spring model includes the plastic deformation behavior of particles in the contact mechanics model [16]. The Edinburgh Elasto-Plastic Adhesion Model (EEPA) captures the historical dependence and key characteristic behavior of viscous solids [17]. According to different soil characteristics, selecting the appropriate contact model is the premise of accurate modeling. To accurately reproduce the physical properties of the soil in the DEM, contact parameters (restitution coefficient, static friction coefficient, rolling friction coefficient, etc.) between materials need to be determined, which have a significant impact on the simulation results. However, it is very difficult to measure these parameters directly [18]. In many studies, the input parameters of DEM are determined by virtual calibration through angle of repose (AoR) tests [19,20,21].

In the field of terramechanics, Bekker’s and Janosi’s models are commonly used to describe soil mechanical properties and then investigate vehicle maneuverability [22,23,24]. The above two semi-empirical models need to obtain load-sinkage curves and torque-angular displacement curves through soil pressure and shear tests, and further obtain soil pressure and shear mechanical characteristics parameters. In traditional research, the penetration and shearing rates allow adjustment in the range of 0 to 10 cm/s [25,26]. Obviously, the loading rate of the tests is far from the running speed of the vehicle on soft ground (about 10 m/s). Therefore, the question of whether the experimental soil mechanical property parameters are suitable for the wheel-soil interaction model under high-speed vehicle conditions is questionable. Under the existing experimental conditions, it is very difficult to achieve high-speed loading under small displacement, so the use of discrete element simulation has become an alternative method.

In this study, the contact parameters of beach sand were calibrated by the optimization design methods. On this basis, the penetration and shear simulation models were constructed by using the DEM method to study the pressure and shear failure mechanism of beach sand under high-speed conditions. This study can provide a theoretical basis for the construction of soil high-speed pressure shear models and the study of wheel-soil interaction.

## 2. Calibration of Discrete Element Parameters

### 2.1. Physical Experiments of AoR

**Figure 1:**
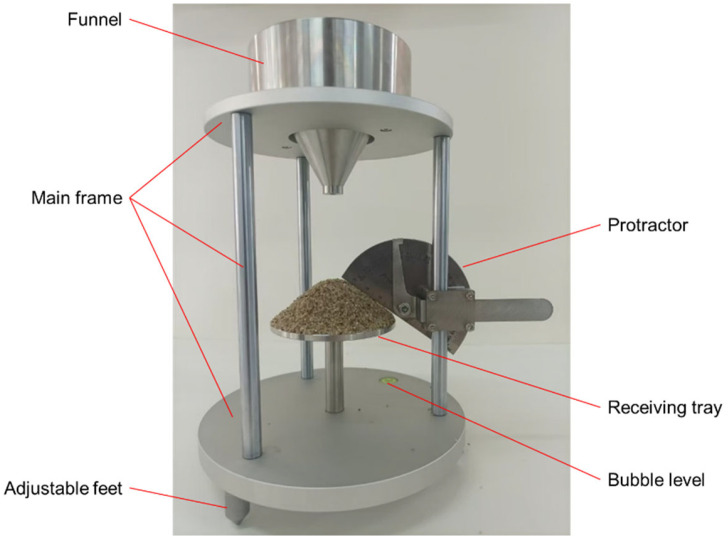
AoR measuring device.

In this study, the AoR of beach sand was measured using an AoR measuring device. The device consists of a main frame, funnel, protractor, receiving tray, adjustable feet, bubble level, etc., as shown in Figure 1. The funnel is fixed to the main frame, and the sand falls onto the receiving tray through the funnel. As the amount of sand gradually increases, a sand pile is formed on the receiving tray, and the angle between the sand pile and the receiving tray is the AoR, which can be measured using a protractor on the device.

To reduce the experimental error of the AoR, the physical experiments were repeated 5 times. The obtained AoR were 36.5°, 37.2°, 36.8°, 36.1°, and 36.9°, respectively. Then the mean value of 36.7° was taken as the AoR of the beach sand.

### 2.2. DEM Simulation Methodology

#### 2.2.1. Selecting the Contact Model

**Figure 2:**
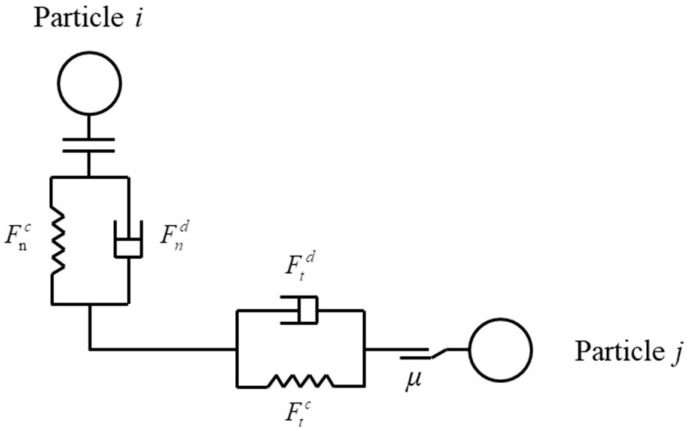
Schematic of simplified Hertz–Mindlin contact model.

Beach sand is a non-cohesive granular material, and the adhesion between particles can be neglected. Therefore, the Hertz–Mindlin (no-slip) contact model is suitable [27,28]. The model simplifies the tangential and normal contact forces as parallel connections of springs and dampers. Figure 2 illustrates the schematic of the contact model between two particles.

The contact force between particle *i* and *j* is decomposed into normal force $F_{n}^{c}$ and tangential force $F_{t}^{c}$. To show the energy absorption between particles, normal damping force $F_{n}^{d}$, and tangential damping force $F_{t}^{d}$ are added to the contact model.

The contact force and damping force are given by [29]

(1) $$F_{n}^{c}=\frac{4}{3}E^{*}{\left(R^{*}\right)}^{1/2}\delta_{n}^{3/2}$$

(2) $$F_{t}^{c}=K_{t}\delta_{t}$$

(3) $$F_{n}^{d}=2\cdot \sqrt{\frac{5}{6}}\frac{\ln e}{\sqrt{\ln^{2}e+\pi^{2}}}\sqrt{K_{n}m^{*}}\overset{\cdot }{v_{n}^{r}}$$

(4) $$F_{t}^{d}=2\cdot \sqrt{\frac{5}{6}}\frac{\ln e}{\sqrt{\ln^{2}e+\pi^{2}}}\sqrt{K_{t}m^{*}}\overset{\cdot }{v_{t}^{r}}$$

where $\delta_{n}$ and $\delta_{t}$ are the normal and tangential overlap, respectively. *e* is the restitution coefficient. *K_n_* and *K_t_* are the normal stiffness and tangential stiffness, respectively, and can be defined as

(5) $$K_{n}=\frac{4}{3}E^{*}\sqrt{R^{*}\delta_{n}}$$

(6) $$K_{t}=8G^{*}\sqrt{R^{*}\delta_{t}}$$

where E* is Young’s modulus, G* is equivalent shear modulus, and R* is equivalent radius.

In DEM simulation, the tangential force can be expressed as ${\mu F}_{n}^{c}$, where *μ* is the static friction coefficient. The additional moment (Ma) caused by rolling friction can be calculated as

(7) $$M_{a}=-\gamma F_{n}^{c}R^{*}$$

where *γ* is the dynamic friction coefficient.

The moment *M* caused by the tangential contact force is given by [30]

(8) $$M=R^{*}\cdot (F_{t}^{c}+F_{t}^{d})$$

Then the resultant moment (*M_r_*) is calculated as

(9) $$M_{r}=M+M_{a}$$

The motion of discrete particles is governed by Newton’s laws of motion. The translational and rotational motion equations of particle *i* are as follows:

(10) $$m_{i}\frac{dv_{i}}{dt}=m_{i}g+F_{n}^{c}+F_{t}^{c}+F_{n}^{d}+F_{t}^{d}$$

(11) $$I_{i}\frac{d\omega_{i}}{dt}=M_{i}$$

where *m_i_* is the mass of particle *i*, *v_i_* is the velocity of particle *i*, *t* is the time, and *g* is the gravitational acceleration. *I_i_* is the inertial moment of particle *i*, *ω_i_* is the angular velocity, and *M_i_* is the resultant tangential torque.

#### 2.2.2. DEM Simulation Setup of Bulk AoR

**Figure 3:**
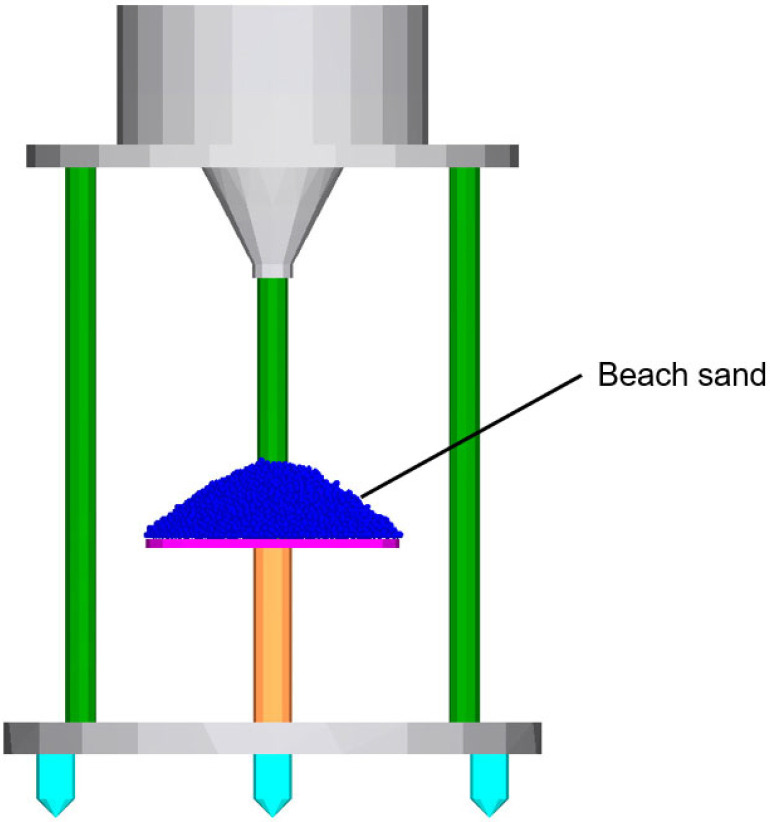
The simulation setup for AoR in EDEM.

In this study, the discrete element model is constructed by using EDEM 2022 software. Firstly, construct a virtual device in the simulation that is identical to the physical experiment, as shown in Figure 3. Then, randomly generate beach sand particles with a particle size of 0.8–1.2 mm to fill the funnel. The beach sand particles are deposited on the receiving tray, forming a sand pile. After all particles reach a stable state, the AoR of the sand pile is finally measured.

In this study, the Plackett–Burman (PB) test was used to screen for factors that have a significant impact on the AoR. Then, the Box Behnken (BB) test was used to obtain the regression model of the significant parameters and obtain the optimal solution. Finally, the optimal parameter values are used for DEM simulation, and error analysis is conducted by comparing the experimental and simulation AoR.

The intrinsic parameters of beach sand and steel are shown in Table 1.

### 2.3. DEM Parameter Calibration of Beach Sand

#### 2.3.1. PB Experiment

In this study, an eleven-factor tables were used to conduct PB tests, with intrinsic parameters and contact parameters of particles as variables, and three virtual parameters were added. Each factor has two levels of high and low, and the parameter range is shown in Table 2.

Table 3 shows the results of the PB test, with a total of 12 tests. The AoR range obtained from the simulation is 20.1–59.3°.

Table 4 lists the effects of different DEM parameters on AoR. It can be seen that the AoR was strongly affected by the static friction coefficient of beach sand-beach sand (*μ*_1_), the dynamic friction coefficient of beach sand-beach sand (*γ*_1_), and the static friction coefficient of beach sand-steel (*μ*_2_). The Poisson’s ratio of beach sand contributes the least to the AoR.

#### 2.3.2. BB Experiment

According to the PB test results, *μ*_1_, *γ*_1,_ and *μ*_2_ have the greatest impact on the AoR. Combined with the steepest ascent test, the parameter range of the BB test was determined, as shown in Table 5.

Results of the Box–Behnken simulation are shown in Table 6.

According to the simulation results, a quadratic regression model for the AoR and significant variables is established.

(12) $$\begin{array}{c} AoR=33.73+4.11\mu_{1}+2.79\gamma_{1}+2.17\mu_{2}-\mu_{1}\gamma_{1}-0.63\mu_{1}\mu_{2}+1.43\gamma_{1}\mu_{2}-3.22\mu_{1}^{2}\\ -2.37\gamma_{1}^{2}+0.0583\mu_{2}^{2} \end{array}$$

**Figure 4:**
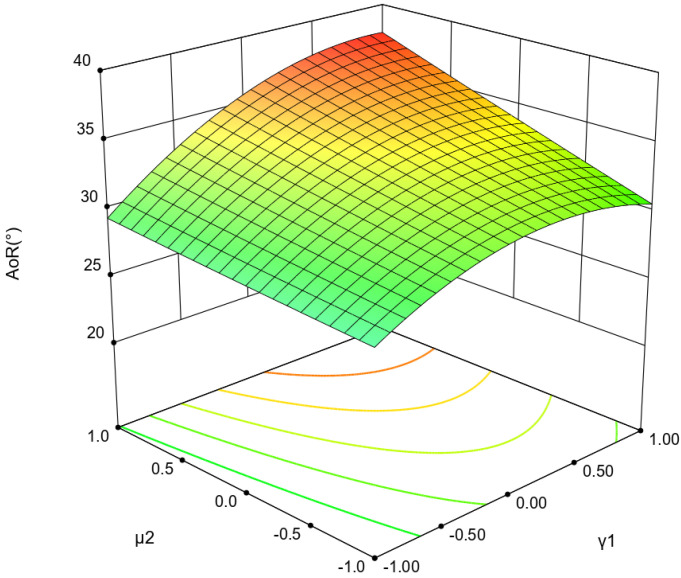
The response surface plot.

As shown in Table 7, the *p*-value of the regression model indicates that there is a significant relationship between the dependent variable and independent variables (*p* = 0.0008 < 0.01). Both the *μ*_1_ and *γ*_1_ have significant effects on the AoR (*p* < 0.01). Moreover, the interaction effects of *γ*_1_ and *μ*_2_ also contribute to the AoR significantly (*p* = 0.0421 < 0.05). Furthermore, the effect of *γ*_1_ on the AoR is more significant than that of *μ*_2_ in the response surface of AoR (Figure 4). The coefficient of determination (R^2^ = 0.9822) and the adjusted coefficient of determination (R_2adj_ = 0.9502) indicate that the regression model is highly reliable.

Taking the actual AoR of beach sand as the response value, the regression model was solved within the factor range. The optimal combination of parameters are *μ*_1_ = 0.41, *γ*_1_ = 0.11, *μ*_2_ = 0.39, respectively. By using these optimal DEM parameters, the simulation AoR (35.5°) was obtained. The relative error with the physical experiment is 3.27%, which proves the feasibility of this method in the calibration of beach sand particles.

## 3. Failure Mechanism of High-Speed Pressure and Shear of Beach Sand

Soil shear failure occurs under the action of wheels, and the reaction force of the soil to the wheels determines the traction performance of vehicles. Therefore, the study of soil pressure shear failure mechanisms is beneficial to improve the traction performance of vehicles.

In the penetration tests, the normal pressure acting on the plate and sinkage are measured to predict the normal pressure distribution on the vehicle–terrain interface. Assuming that the soil is uniform, Bekker proposed an empirical model [33].

(13) $$p=(\frac{k_{c}}{b}+k_{\varphi })z^{n}=Kz^{n}$$

where *p* is the pressure applied to the plate, *z* is the sinkage, *K* is the sinkage modulus, and *n* is the sinkage exponent.

In addition to the pressure-sinkage model, another basic model for studying wheel-soil interaction is the shear stress-displacement model. The shear stress-displacement relationship is used to determine the tangential interaction stress at wheel-soil interfaces. The integral of tangential interaction stress provides traction for the vehicle. Janosi and Hanamoto’s shear stress-displacement equation is as follows [34].

(14) $$\tau =(c+\sigma \tan\varphi )\left(1-e^{\frac{-j}{K}}\right)$$

where *τ* is the shear stress, *c* is the soil cohesion, *σ* is the normal stress, *φ* is the angle of internal friction, *j* is the slip displacement, and *K* is the soil deformation coefficient.

Assuming that a constant shearing stress acts on the anulus area, the total moment on the annular ring is given by

(15) $$T=\frac{2\pi \tau (r_{1}^{3}-r_{2}^{3})}{3}$$

where *r*_1_ is the outside diameter of the annular ring, and *r*_2_ is the inside diameter of the annular ring.

**Figure 5:**
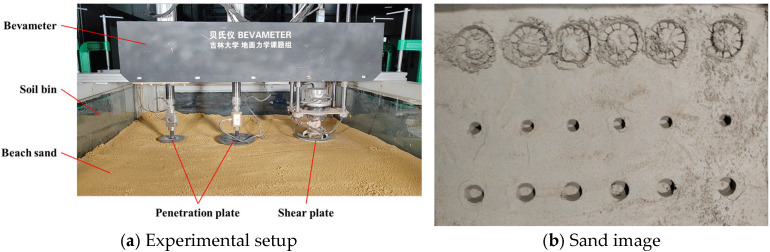
Penetration and shear test.

**Figure 6:**
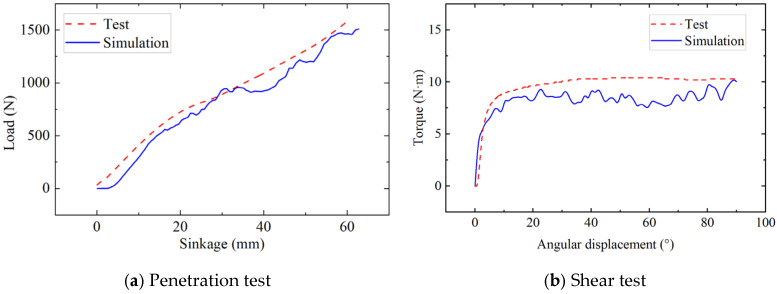
Comparison of test and simulation.

Based on Bekker’s and Janosi’s model, a bevameter was developed for measuring the terramechanics characteristics. The bevameter is mainly composed of two penetration plates and a shear plate (Figure 5a). Pressure and displacement sensors connected to the press plate are used to obtain pressure sinkage curves. A torque angle sensor connected to the shear plate is used to obtain the shear torque-angular displacement curve of beach sand. The penetration plate and sensors are connected with the round-trip linear electric motor, and the shear plate and torque sensor are integrated with the harmonic deceleration servo motor. Before the experiment, the bevameter frame is fixed on the soil bin, and then the pressure and shear curves can be obtained by controlling the motor downward or rotating. In the experiment, the penetration rate is 3 mm/s, and the shear rate is 17.3 mm/s. To reduce the random error, the mean value of 5 repeated experiments was taken as the experimental result. Figure 5 shows the experimental scene of the bevameter and the sand image after the experiment. To verify the accuracy of DEM parameters of beach sand, the DEM models of press and shear plate are constructed and compared with physical experiment results, as shown in Figure 6. The average error of the press plate simulation is 10.3%, and that of the shear plate simulation is 12.6%, indicating a high degree of consistency between simulation and experiment.

**Figure 7:**
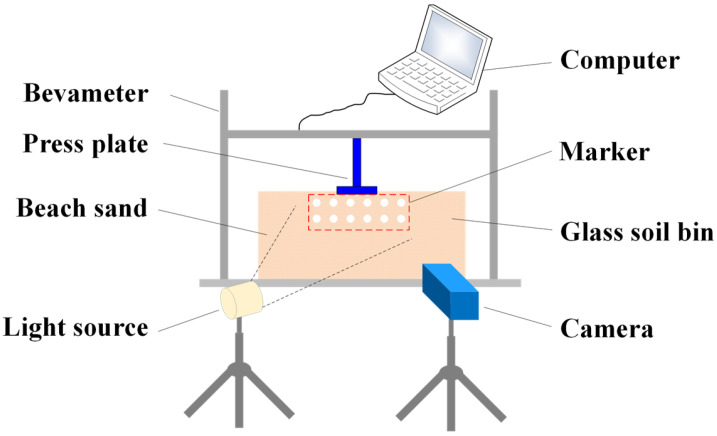
Failure experiment.

**Figure 8:**
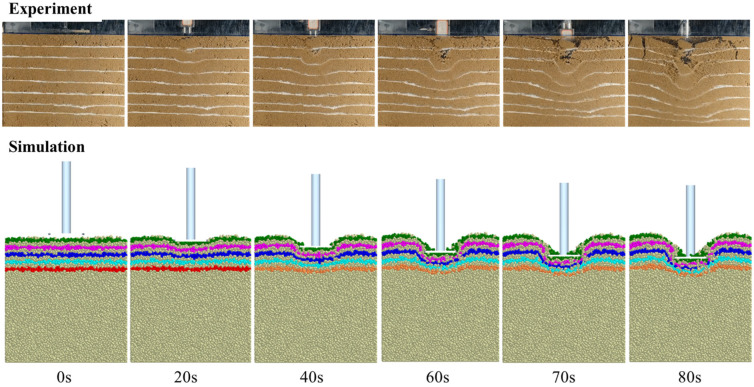
Comparison between the experiment and simulation.

To study the soil failure process of beach sand, an experimental system for soil failure was set up, as shown in Figure 7. The system consists of beach sand, a marker, a Bevameter, a glass soil bin, a light source, a camera, and a computer. In the experiment, the sand was marked by layers of lime, and then the pressure plate experiment was carried out. At the same time, the failure process was recorded by a camera. Figure 8 shows the comparison between the experiment and simulation of the beach sand failure process. In the simulation, the beach sand is stratified every 10 mm along the downward pressure direction, and different color attributes are set to observe the specific failure of the sand. It can be seen that with the downward movement of the pressure plate, the soil particles under the pressure plate move downward in a layered manner, and the particle layer is clearly visible. The sand particles move down and around the press plate, and the failure sliding surface of the sand is approximately an inclined plane. Moreover, the simulation results are highly consistent with the experimental results.

In this study, the simulation models of penetration plate and shear plate tests were established by using the DEM parameters obtained from the calibration, and the beach sand high-speed pressure and shear failure mechanism were studied.

**Figure 9:**
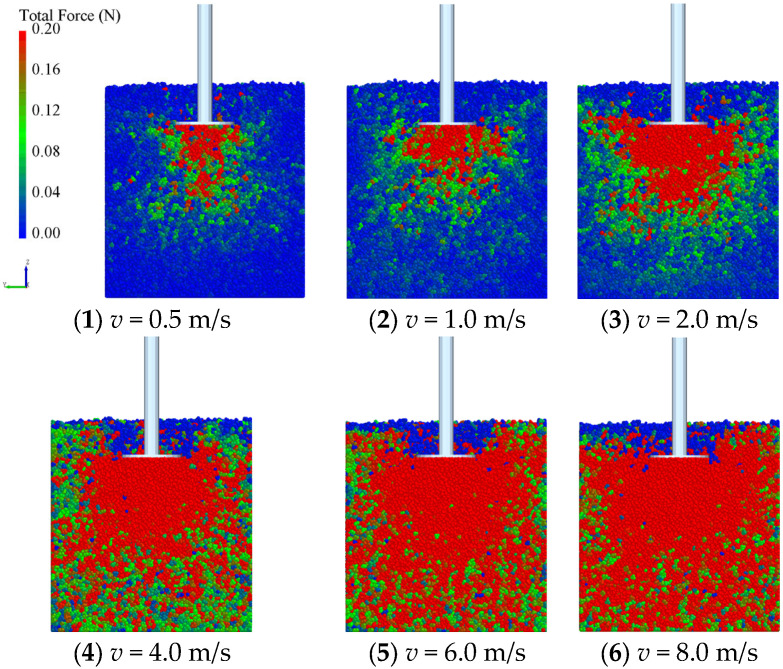
Particle velocity field of penetration simulation.

In the penetration simulations, the diameter of the sinkage plate is 100 mm, and the penetration rates are 0.5 m/s, 1.0 m/s, 2.0 m/s, 4.0 m/s, 6.0 m/s, and 8.0 m/s, respectively. Figure 9 shows the stress of sand particles under different penetration rates. With the increase of the penetration rate, the influence area of the sinkage plate also gradually increases. This indicates that when the penetration rate increases, the disturbance range to the soil is obviously expanded. At the same time, the stress of sand particles increases, which means that the reaction force of particles to the sinkage plate also increases, indicating that the soil bearing capacity increases at high speed.

**Figure 10:**
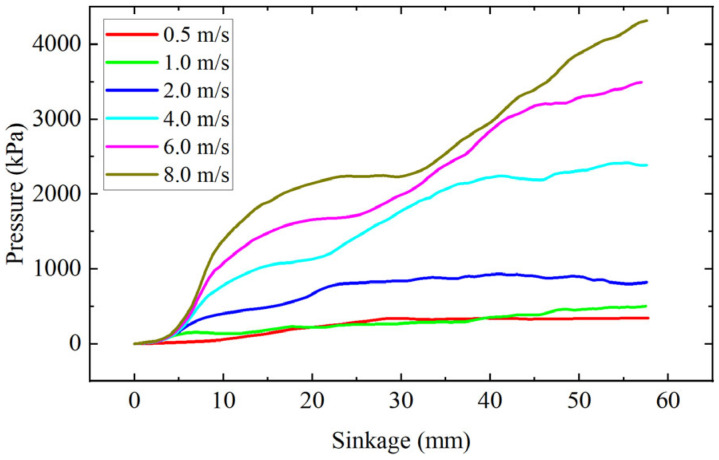
Pressure-sinkage curve.

Figure 10 shows the pressure-sinkage simulation results under different penetration rates. It can be seen from the figure that there are obvious differences in the pressure sinkage curves obtained under different penetration rates. For the same sinkage depth (57 mm), when the penetration rate is 0.5 m/s, the pressure is 342.3 kPa, while when the penetration rate is 8 m/s, the pressure is 4316.7 kPa, which is 12.7 times that of the penetration rate of 0.5 m/s. This indicates that the bearing capacity of soil increases with the increase in penetration rate.

**Figure 11:**
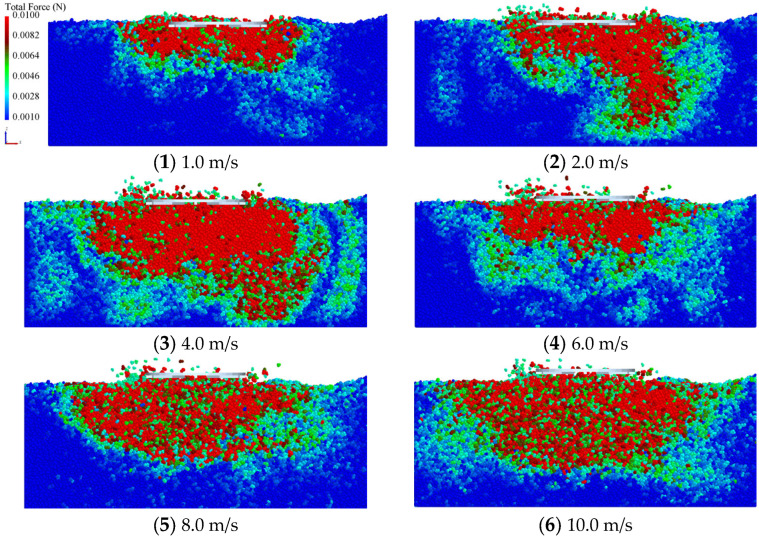
Particle velocity field of shear plate simulation.

In the shear simulation, the outer diameter of the annular shear plate is 200 mm, the inner diameter is 100 mm, and twelve 5 mm high spikes are uniformly distributed around the plate. The shear rates are 1.0 m/s, 2.0 m/s, 4.0 m/s, 6.0 m/s, 8.0 m/s, 10.0 m/s, respectively. Figure 11 is the stress nephogram of sand particles at different shear rates. It can be seen from the figure that with the increase of shear rate, the circumferential and vertical disturbance areas of the sand increase, and the total force of disturbed particles increases.

**Figure 12:**
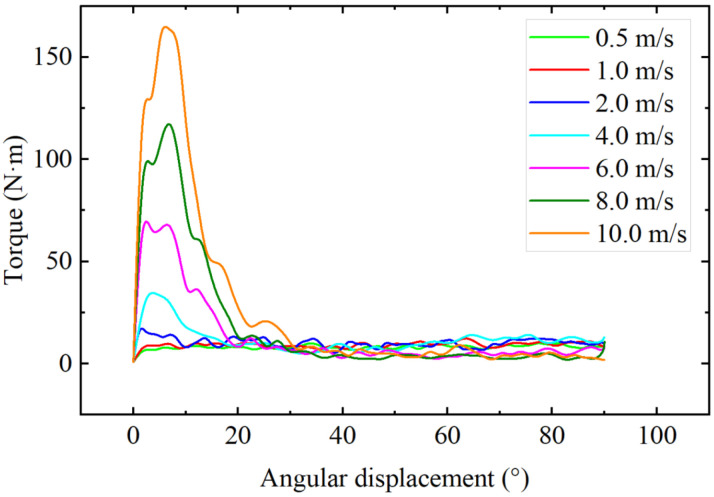
Shear torque.

Figure 12 shows the shear angle-torque simulation results at different shear rates. In the initial shear stage (angle displacement 0~30°), the maximum shear torque increases with the increase of shear rate. The maximum shear torque at 10.0 m/s is 19.3 times that of 0.5 m/s, which is mainly due to the inertia effect of the high-speed start of the shear plate. In the stable shear stage (angle displacement 30~90°), the average shear torque initially increases slightly with the increase of speed, and then decreases significantly when the speed exceeds 4 m/s. This is because as the shear rate increases, the disturbance of the soil by the shear plate increases, the velocity of soil particles increases, resulting in a decrease in the number of soil particles in contact with the shear plate, thereby reducing the shear torque.

## 4. Discussion

Under dynamic loading conditions, the mechanical property parameters of the soil may have significant differences from those under quasi-static loading conditions. This will affect the interaction between the wheels and the soil, thereby influencing the accuracy of the vehicle traversability evaluation model. In this study, the DEM contact parameters of the beach sand were first calibrated. Then, the simulation models of penetration and shear were constructed using the calibrated parameters to study the failure mechanism of the beach sand under high-speed penetration and shear conditions.

The pressure plate penetration test, as a common testing method for soil pressure-bearing characteristic parameters, has a penetration rate usually less than 10 cm/s [25,26]. When a vehicle is traveling at high speed, the vertical component of the wheel speed may exceed 8 m/s [35]. Obviously, the low-speed penetration test did not take into account the effect of velocity, resulting in significant limitations of the obtained soil pressure-bearing parameters when applied to high-speed wheel-soil interactions. It can be seen from Figure 9 that with the increase of penetration speed, the contact force between soil particles increases significantly. At high speed, the distribution range of the force chain between particles is small, and the force value is small. At high speed, the force chain distribution is more extensive, and the maximum contact force increases significantly. At high speed, the force chain distribution is more extensive, and the maximum contact force increases significantly. This change in force characteristics directly reflects the variation of the dynamic bearing properties of the soil; with the increase of penetration speed, the collision frequency between particles increases, and the kinetic energy transfer efficiency enhances, resulting in a significant increase in the reaction force exerted by particles on the penetration plate, as shown in Figure 10. Then, when the interaction speed between the wheels and the soil increases, the bearing capacity of the ground may also increase significantly, which may affect the sinkage of the wheels and thereby influence the driving resistance.

Similar to the penetration test, the shear rate of the traditional shear test is generally less than 10 cm/s, while the shear rate of the vehicle on the soil exceeds 10 m/s. It can be seen from Figure 11 that with the increase of shear rate, the total force of disturbed particles increases. As shown in Figure 12, in the stable shear stage (angle displacement 30–90°), the shear torque increases slightly at first and then begins to decrease as the shear speed increases. This change is closely related to the dynamic response on the shear plate; the increase in shear rate leads to an increase in the shear strength of the soil, but when the shear speed continues to increase, due to the existence of an upward impact force component, the vertical load on the shear plate decreases, resulting in a decrease in shear torque. This phenomenon suggests that the driving speed may affect the shear force during vehicle operation, thereby influencing the vehicle’s traction performance.

## 5. Conclusions

In this paper, a set of DEM parameters of beach sand was obtained through parameter calibration. It was found that the static friction coefficient and rolling friction coefficient of beach sand have significant effects on the AoR. The AoR simulation was conducted using the DEM parameters obtained from calibration as input, with a simulation error of 3.27%, proving that the method has high accuracy for sand DEM parameter calibration. Then, penetration and shear simulation models were established, and the high-speed pressure and shear failure mechanism of beach sand was studied. Our results show that the bearing capacity of the beach sand increased significantly with the increase in the penetration rate. In the stable shear stage, the average shear torque initially increases slightly with the increase of speed and then decreases when the speed exceeds 4 m/s, indicating that the increase of speed has no significant effect on the shear torque. In future studies, we hope to further explore the effect of velocity on wheel-soil interaction. Our relevant research findings can be utilized in the design of locomotion systems, traversability evaluation, and path planning of planetary exploration vehicles, off-road vehicles, and agricultural vehicles.

## Figures and Tables

**Table 1:** Material parameters.

Material	Young’s Modulus [Pa]	Poisson’s Ratio	Density	Reference
Beach sand	5 × 10^7^	0.3	2653	[31]
Steel	1.849 × 10^11^	0.3	7850	[32]

**Table 2:** Parameters of the PB experiment.

Parameters	Symbol	Low Level (−1)	High Level (+1)
Poisson’s ratio of beach sand	*ν*	0.25	0.35
Young’s modulus of beach sand/MPa	*E*	30	70
Beach sand-beach sand coefficient of restitution	*e*_1_	0.3	0.7
Beach sand-beach sand static friction coefficient	*μ*_1_	0.1	0.9
Beach sand-beach sand dynamic friction coefficient	*γ*_1_	0.01	0.3
Beach sand-steel coefficient of restitution	*e*_2_	0.3	0.7
Beach sand-steel static friction coefficient	*μ*_2_	0.1	0.9
Beach sand-steel dynamic friction coefficient	*γ*_2_	0.01	0.3

**Table 3:** The result of the PB experiment.

No.	*ν*	*E*		*e*_1_	*μ*_1_		*γ*_1_	*e*_2_		*μ*_2_	*γ*_2_	AoR (°)
1	1 (0.35)	−1 (30)	1	1 (0.7)	1 (0.9)	−1	−1 (0.01)	−1 (0.3)	1	−1 (0.1)	1 (0.3)	29.8
2	−1 (0.25)	1 (70)	1	1	−1 (0.1)	−1	−1 (0.3)	1 (0.7)	−1	1 (0.9)	1	27.2
3	−1	−1	−1	−1 (0.3)	−1	−1	−1	−1	−1	−1	−1 (0.01)	20.1
4	1	1	−1	1	1	1	−1	−1	−1	1	−1	35.3
5	1	1	−1	−1	−1	1	−1	1	1	−1	1	21.3
6	1	−1	−1	−1	1	−1	1	1	−1	1	1	59.3
7	1	−1	1	1	−1	1	1	1	−1	−1	−1	22.2
8	1	1	1	−1	−1	−1	1	−1	1	1	−1	33.2
9	−1	1	−1	1	1	−1	1	1	1	−1	−1	53.1
10	−1	1	1	−1	1	1	1	−1	−1	−1	1	49.3
11	−1	−1	−1	1	−1	1	1	−1	1	1	1	31.9
12	−1	−1	1	−1	1	1	−1	1	1	1	−1	35.8

**Table 4:** Analysis of the significance of parameters in the PB experiment.

Parameters	Effect	Sum of Squares	Contribution/%	Significance
*ν*	−2.72	22.14	1.23	8
*E*	3.38	34.34	1.91	4
*e*_1_	−3.25	31.69	1.76	5
*μ*_1_	17.78	948.74	52.78	1
*γ*_1_	13.25	526.69	29.3	2
*e*_2_	3.22	31.04	1.73	6
*μ*_2_	4.48	60.3	3.35	3
*γ*_2_	3.18	30.4	1.69	7

**Table 5:** Factors and levels for the BB experiment.

Variables	Low Level	Middle Level	High Level
*μ*_1_	0.1	0.3	0.5
*γ*_1_	0.01	0.08	0.15
*μ*_2_	0.1	0.3	0.5

**Table 6:** Results of the BB experiment.

No.	*μ*_1_	*γ*_1_	*μ*_2_	AoR (°)
1	−1 (0.1)	0 (0.08)	−1 (0.1)	23.1
2	0 (0.3)	−1 (0.01)	1 (0.5)	28.5
3	0	−1	−1	27.5
4	1 (0.5)	0	−1	33.2
5	−1	0	1	29.2
6	1	0	1	36.8
7	0	0	0 (0.3)	33.0
8	1	1 (0.15)	0	33.1
9	−1	−1	0	21.2
10	0	0	0	34.4
11	0	1	−1	31.5
12	0	1	1	38.2
13	0	0	0	33.8
14	1	−1	0	30.8
15	−1	1	0	27.5

**Table 7:** ANOVA of the quadratic model for BB experiment.

Source	Sum of Squares	df	Mean Square	F Value	*p*-Value Prob > F	
Model	304.66	9	33.85	30.67	0.0008	significant
*μ*_1_	135.30	1	135.30	122.57	0.0001	
*γ*_1_	62.16	1	62.16	56.31	0.0007	
*μ*_2_	37.84	1	37.84	34.29	0.0021	
*μ*_1_ *γ*_1_	4.00	1	4.00	3.62	0.1153	
*μ*_1_ *μ*_2_	1.56	1	1.56	1.42	0.2876	
*γ*_1_ *μ*_2_	8.12	1	8.12	7.36	0.0421	
*μ*_1_^2^	38.20	1	38.20	34.61	0.0020	
*γ*_1_^2^	20.68	1	20.68	18.74	0.0075	
*μ*_2_^2^	0.0126	1	0.0126	0.0114	0.9192	
Residual	5.52	5	1.10			
Lack of Fit	4.53	3	1.51	3.06	0.2558	not significant
Pure Error	0.9867	2	0.4933			
Cor Total	310.18	14				

## Data Availability

The original contributions presented in this study are included in the article. Further inquiries can be directed to the corresponding authors.
